# Autologous bone marrow mononuclear cell transplantation in patients with chronic traumatic brain injury- a clinical study

**DOI:** 10.1186/s13619-020-00043-7

**Published:** 2020-06-02

**Authors:** Alok K. Sharma, Hemangi M. Sane, Pooja P. Kulkarni, Nandini Gokulchandran, Hema Biju, Prerna B. Badhe

**Affiliations:** 1 Department of Medical Services, NeuroGen Brain & Spine Institute, Plot 19, Sector 40, Next to Seawood Grand Central Station (W), Off Palm Beach Road, Nerul, Navi Mumbai, 400706 India; 2 Department Of Research & Development, NeuroGen Brain & Spine Institute, Plot 19, Sector 40, Next to Seawood Grand Central Station [W], Off Palm Beach Road, Nerul, Navi Mumbai, 400706 India; 3 Department of Neurorehabilitation, NeuroGen Brain & Spine Institute, Plot 19, Sector 40, Next to Seawood Grand Central Station [W], Off Palm Beach Road, Nerul, Navi Mumbai, 400706 India; 4 Department of Regenerative Laboratory Services, NeuroGen Brain & Spine Institute, Plot 19, Sector 40, Next to Seawood Grand Central Station [W], Off Palm Beach Road, Nerul, Navi Mumbai, 400706 India

**Keywords:** Autologous, Bone marrow, Mononuclear cells, Chronic, Traumatic brain injury, PET CT scan, Neurorehabilitation

## Abstract

**Background:**

Chronic Traumatic Brain Injury (TBI) is one of the common causes of longterm disability worldwide. Cell transplantation has gained attention as a prospective therapeutic option for neurotraumatic disorders like TBI. The postulated mechanism of cell transplantation which includes angiogenesis, axonal regeneration, neurogenesis and synaptic remodeling, may tackle the pathology of chronic TBI and improve overall functioning.

**Methods:**

To study the effects of cell transplantation, 50 patients with chronic TBI were enrolled in an open label non-randomized study. The intervention included intrathecal transplantation of autologous bone marrow mononuclear cells and neurorehabilitation. Mean follow up duration was 22 months. Fifteen patients underwent second dose of cell transplantation, 6 months after their first intervention. Percentage analysis was performed to analyze the symptomatic improvements in the patients. Functional independence measure (FIM) was used as an outcome measure to evaluate the functional changes in the patients. Statistical tests were applied on the pre-intervention and post-intervention scores for determining the significance. Comparative Positron Emission Tomography- computed tomography (PET CT) scans were performed in 10 patients to monitor the effect of intervention on brain function. Factors such as age, multiple doses, time since injury and severity of injury were also analyzed to determine their effect on the outcome of cell transplantation. Adverse events were monitored throughout the follow up period.

**Results:**

Overall 92% patients showed improvements in symptoms such as sitting and standing balance, voluntary control, memory, oromotor skills lower limb activities, ambulation, trunk & upper limb activity, speech, posture, communication, psychological status, cognition, attention and concentration, muscle tone, coordination, activities of daily living. A statistically significant (at *p* ≤ 0.05 with *p*-value 0) improvement was observed in the scores of FIM after intervention on the Wilcoxon signed rank test. Better outcome of the intervention was found in patients with mild TBI, age less than 18 years and time since injury less than 5 years. Ten patients who underwent a repeat PET CT scan brain showed improved brain metabolism in areas which correlated to the symptomatic changes. Two patients had an episode of seizures which was managed with medication. They both had an abnormal EEG before the intervention and 1 of them had previous history and was on antiepileptics. No other major adverse events were recorded.

**Conclusion:**

This study demonstrates the safety and efficacy of cell transplantation in chronic TBI on long term follow up. Early intervention in younger age group of patients with mild TBI showed the best outcome in this study. In combination with neurorehabilitation, cell transplantation can enhance functional recovery and improve quality of life of patients with chronic TBI. PET CT scan brain should be explored as a monitoring tool to study the efficacy of intervention.

## Background

Traumatic brain injury (TBI) remains the most common cause of trauma related deaths and disability especially amongst the young individuals (Roozenbeek et al., 2013). Its outcome is determined by two different mechanisms/stages. Primary insult that occurs at the moment of the accident/impact and secondary insult which constitutes the consecutive pathological processes (Prins et al., 2013). The effects of a traumatic brain injury on an individual depends on the type, area of the brain involved and severity of injury. The symptomatic representations are heterogenous and can range from physical effects such as balance and postural problems, headaches and dizziness to cognitive, emotional and behavioral disturbances such as memory problems and anger (Fleminger & Ponsford, 2005).

Acute phase of TBI involves the immediate damage produced from the insult, and the chronic phase may extend for years. Chronic TBI is characterised by inflammation, cell death, and neural dysfunction activated by the primary insult (McKee & Daneshvar, 2015; Werner & Engelhard, 2007). Alterations in cerebral blood flow and oxygenation, edema, excitotoxicity, cell death, disruption of the blood brain barrier, and generalized atrophy is also commonly observed (Acosta et al., 2015a).

There have been conventional treatments such as surgical intervention, physical and behavioral therapy, Hyperbaric Oxygen therapy (HBOT), and medical management of associated conditions (Bennett et al., 2012; Xiong et al., 2009). These are focused on reducing the consequences of secondary insult in acute TBI. Due to the brain’s limited capacity to regenerate the damaged neurons in chronic injury, cell transplantation may be a useful treatment strategy. In past, few years, it has gained significant attention as a prospective therapeutic option for various neurological disorders (Sharma et al., 2012; Sharma et al., 2013a; 2013b; Zhang et al., 2013). Different cell types such as bone marrow derived cells, neural stem cells, embryonic cells, umbilical cord blood derived cells, induced pluripotent cells (iPSCs), etc. have been explored (Ul Hassan et al., 2009; Liu et al., 2000; Muotri, 2010; Zhao et al., 2002). The postulated mechanism of cell transplantation includes neuroprotection, neurogenesis, angiogenesis, synaptic remodeling and axonal regeneration which may help repair the neuronal damage in chronic TBI (Xiong et al., 2010).

To assess the safety and feasibility of cell transplantation in chronic TBI, we had previously conducted a pilot study on a small population (*N* = 14) of patients (Sharma et al., 2015). The current study is an extension with larger sample size of 50 chronic TBI patients and a long-term follow up to further establish the efficacy of intervention.

## Materials and method

### Ethics statement

Selection of patients included in this study was based on the criteria of World Medical Association Helsinki Declaration for Ethical Principles for medical research involving human subjects (World Medical Association, 2013). The protocol of the study was reviewed and approved by the Institutional Ethics Committee. The treatment plan was explained to the patients/ caretakers in detail along with possible adverse events. A written informed consent was obtained either from the patients or their families depending on the patients’ cognitive status.

### Study design and patient selection

An open label non-randomized clinical study was conducted to evaluate the safety and efficacy of intrathecal administration of autologous bone marrow mononuclear cells (BMMNCs) in chronic traumatic brain injury. The study was conducted in a single hospital centre and included 50 patients with chronic TBI.

*Inclusion criteria*: Diagnosed cases of chronic TBI with age above 1 year and patients who were hemodynamically stable were included in the study.

*Exclusion criteria*: The patients were excluded if there was presence of any malignancy, severe liver dysfunction, renal failure, any bone marrow disorder, HIV/HBV/HCV, bleeding tendency, severe anemia [Hb < 9], other acute medical conditions such as respiratory infection, other acute infections and pyrexia and pregnancy or lactation.

### Intervention

The intervention included a combination of cell transplantation and neurorehabilitation. Cell transplantation included administration of autologous BMMNCs intrathecally and neurorehabilitation included a personalised rehabilitation program based on individual needs which comprised of modalities such as occupational therapy, physiotherapy, aquatic therapy, speech therapy and psychological intervention.

#### Pre-intervention assessment

A detailed neuro-evaluation was done for every patient by medical experts. Along with this, hematological, biochemical and serological tests were also performed. Magnetic Resonance Imaging (MRI) of brain with Diffusion tensor imaging (DTI), Electroencephalography (EEG), and Positron Emission Tomography- Computed Tomography (PETCT) brain scans were done before the intervention in all patients. Functional Independence Measure (FIM) was used to evaluate the functional independence of all the patients.

#### Bone marrow aspiration and isolation of bone marrow mononuclear cells

Seventy-two hours and twenty-four hours prior to the procedure, all the patients were administered with Granulocyte Colony Stimulating Factor (G-CSF) injections for stimulation and mobilization of the bone marrow stem cells. Aspiration of bone marrow was carried out under sedation with local anesthesia. 80–100 mL of bone marrow was aspirated from the anterior superior iliac crest depending on the age and body weight of the patient using the bone marrow aspiration needle. The bone marrow was collected in heparinized tubes. Density gradient method was used to separate BMMNCs from the aspirate. Trypan Blue dye was used to calculate the cell viability which was then confirmed using propidium iodide dye on TALI (Invitrogen). The average number of cells injected were 1.28 × 10^8^ with an average cell viability of 97%. CD34+ count was done by fluorescence activated cell sorting (FACS) using CD34 PE antibody (BD Biosciences) and the average count was found to be 256.34 cells/uL.

#### Administration of bone marrow mononuclear cells

Autologous BMMNCs were injected immediately after separation intrathecally between fourth and fifth lumbar vertebrae using spinal needle. The injection procedure is performed under local anaesthesia with or without mild sedation. At the same time, every patient is administered 20 mg/kg body weight methyl prednisolone in 500 ml Ringer Lactate intravenously in order to enhance survival of the injected cells.

Fifteen patients underwent second dose of cell transplantation, 6 months after their first intervention. Protocol for which remained the same. To evaluate the efficiency of multiple transplantations,

To evaluate the efficiency of multiple transplantations, we performed a comparative analysis between patients who underwent single and two doses of BMMNC transplantation.

#### Neurorehabilitation

Immediately after cell transplantation, every patient underwent a personalized neurorehabilitation program which included, occupational therapy, physiotherapy, psychological therapy, speech therapy and aquatic therapy. The patients were also advised to continue neurorehabilitation after discharge and were given a home program for the same.

### Study analysis

Patients were followed up at regular intervals post intervention and a detailed neurological assessment was performed at follow up.

#### Percentage analysis

Percentage analysis was performed to study improvements in each symptom. Overall improvements were categorised as mild, moderate and significant based on the grading system which we designed for the study. The grading system devised to evaluate the functional outcome in every individual as follows:

- *Mild improvement*: improvement seen in less than 30% symptoms;
- *Moderate improvements*: improvements seen in 30–60% symptoms;
- *Significant improvements*: improvements seen in more than 60% symptoms along with change in FIM scores

#### Outcome measures

FIM was used to monitor changes in functional dependence of patients while performing activities of daily living. It was measured before intervention and at follow up.

#### PET CT scan of the brain

18 F-FDG PET CT was used as a monitoring tool to assess the outcome of cell transplantation in chronic TBI. It was performed before the intervention and a comparative PET was performed 6 months later to study the metabolic changes occurring in the brain due to the intervention. Consent was obtained from 10 patients for repeating PET-CT scan of the brain 6 months after intervention.

### Statistical analysis

Statistical analysis was performed using IBM SPSS version 2.0 software. The difference between pre-intervention and post- intervention FIM scores were compared using Wilcoxon matched-pairs signed rank test to find its significance. The significance level was pre-determined at *p* ≤ 0.05.

#### Factors affecting the outcome of cell transplantation

A detailed subgroup analysis was done to determine the effect of various factors such as age at intervention (< 18 years and > 18 years), number of doses, time since injury and severity of injury based on Glasgow Coma Scale (GCS) on the outcome of cell transplantation.

#### Adverse event monitoring

During the stay in the hospital and after discharge, signs and symptoms of any major or minor adverse events were monitored at regular intervals to establish short-term and long-term safety. Adverse events were grouped as procedure related and cell transplantation related. Fever, spinal headache, nausea, diarrhea, vomiting, pain at the site of aspiration and injection, anesthesia related issues and minor allergic reactions were considered as minor procedure related adverse events which could be managed by medication immediately. Hematoma at the site of aspiration and injection, bleeding at the site of aspiration or injection, local infection at the site of injection or aspiration, meningitis, respiratory distress, major allergic reactions were considered as major procedure related adverse events and new seizures or increased seizure intensity /frequency were considered as major cell transplantation related adverse events.

## Results

### Demographic analysis

#### Description of the sample

The total population included in the study was 50 including 42 (84%) males and 8 (16%) females. The age of the population ranged from 7 to 64 years with a mean age of 29 years (Table 1).

**Table 1:** Demographical data of the patients

Total patients	*n* = 50	
Sex	Male	42
	Female	8
Age	Avg. age (in years)	29.4
	< 18 years	10
	> 18 years	40
Cause of trauma	Road Traffic Accident (RTA)	40
	Others	10
Duration since injury	< 5 years	32
	> 5 years	18
Severity based on CGS	Mild	27
	Moderate	16
	Severe	7
History of seizures	Present	16
	Absent	34

### Percentage analysis

#### Overall improvements based on the grading system

**Fig. 1:**
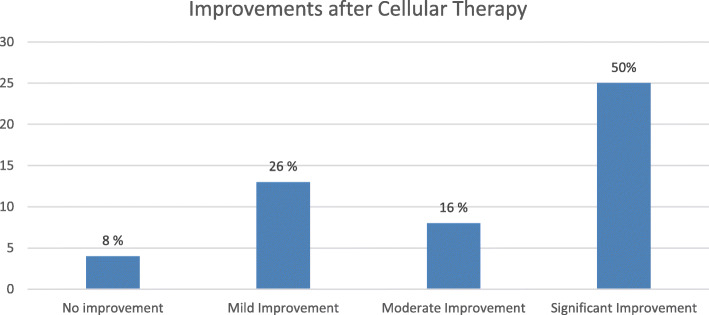
Graph representing overall improvements in TBI after cell transplantation

Overall 90% patients i.e. 40 out of 50 patients showed improvement after cell transplantation. Based on the grading system as described above, 25 (50%) showed significant improvement, 8 (16%) showed moderate improvement, 13 (26%) cases showed mild improvement, 4 (8%) showed no improvement (Fig. 1).

#### Symptomatic improvements

**Fig. 2:**
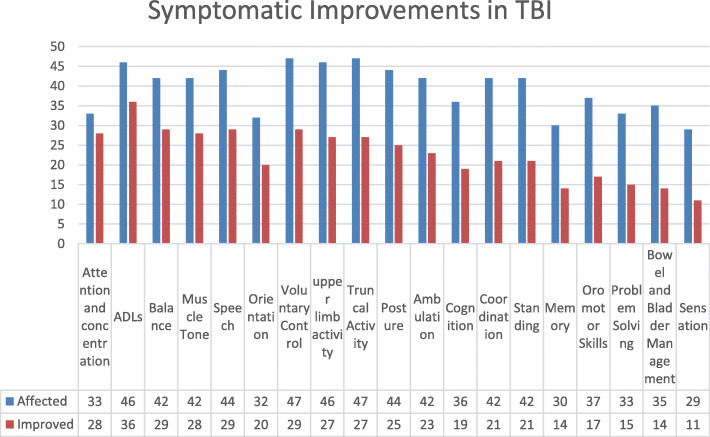
Graph demonstrating symptomatic improvements in patients with chronic TBI after cell transplantation

Average follow up was 22 months (range: 3–71 months). During the symptomatic analysis, patients showed improvement in balance, voluntary control, memory, oromotor skills, lower limb activities, ambulation, trunk & upper limb activity, speech, posture, communication, psychological status, cognition, muscle tone, coordination, activities of daily living (ADLs) (Fig. 2 and Table 2).

**Table 2:** Percentage analysis for each symptomatic improvement in chronic TBI patients

Symptoms	Affected	Improved	Percentage improvement
Attention and concentration	33	28	84.84
ADLs	46	36	78.26
Balance	42	29	69.04
Muscle Tone	42	28	66.66
Speech	44	29	65.9
Orientation	32	20	62.5
Voluntary Control	47	29	61.7
upper limb activity	46	27	58.69
Truncal Activity	47	27	57.44
Posture	44	25	56.81
Ambulation	42	23	54.76
Cognition	36	19	52.77
Coordination	42	21	50
Standing	42	21	50
Memory	30	14	46.66
Oromotor Skills	37	17	45.94
Problem Solving	33	15	45.45
Bowel and Bladder Management	35	14	40
Sensation	29	11	37.93

#### Outcome measures

30 out of 50 (60%) patients showed improved FIM scores after intervention at follow up. On Wilcoxon signed rank test, the change in FIM scores was found to be significant at *p* ≤ 0.05 with *p*-value 0 (Table 3).

**Table 3:** Result of statistical analysis of FIM scores

Scale	Mean score before cellular therapy	Mean Score after cellular therapy	Z Value	Significance
FIM	55.84	62.2	−4.7821	Significant

### PET CT scan brain

On comparing the PET CT scan brain performed before and 6 months after intervention in 10 patients, improved brain metabolism was recorded. It was observed that the metabolic activity in frontal lobe, mesial temporal structures (amygdala, hippocampus), parietal cortex, occipital lobe, thalamus and cerebellum had improved. These changes in the metabolic activity correlated with the clinical and functional improvements demonstrated by these patients (Table 4).

**Table 4:** Areas of brain showing increased metabolism in PET CT scan corresponding to the functional improvements in ten patients

Patients	Area of the brain showing improved metabolism	Symptomatic improvement
1	Temporal, parietal lobe, left mesial temporal, bilateral cingulate, amygdala, cerebellum, basal ganglia	Emotion, attention, cognition, memory, coordination, balance, voluntary motor control, learning, cognition, speech, memory
2	Cerebellum, thalamus, occipital lobe and basal ganglia	coordination, balance, voluntary motor control, learning, Vision and perception, Motor control, sensory functions
3	Frontal lobes, parietal lobes, l occipital lobes.	Vision and perception, Planning, Long term memory, emotions, speech, problem solving, Movement, orientation
4	Parieto-Occipital Areas	Cognition, speech, sensation, orientation and visual perception
5	Cingulate Gyri, Amygdala, Frontal, temporal lobe	Emotional responses, memory, attention, Planning, Long term memory, emotions, speech, problem solving
6	Basal ganglia, temporal cortex, cerebellum, bilateral thalamus	Coordination, balance, voluntary motor control, learning, Motor control, sensory functions
7	Cerebellum, basal ganglia	Coordination, balance, voluntary motor control, learning, Motor control
8	Bilateral cerebellum, right frontal, parietal, temporal and occipital cortex, right basal ganglia and right thalamus	Emotion, attention, cognition, memory, co-ordination, balance, voluntary motor control, learning, cognition, speech, memory, sensory functions
9	Bilateral cerebellum, vermis, cingulate regions, Right Superior parietal gyrus	Voluntary movements, posture, balance, attention span, cognition, emotional response
10	Cingulate cortex, basal ganglia and cerebellum	Emotional responses, memory, problem solving, co-ordination, balance, voluntary motor control,

### Factors affecting the outcome after the intervention

#### Age

On performing age-wise analysis of FIM scores, it was observed that more patients in the age group of < 18 years (paediatric) showed improvement as compared to patients in the age group of > 18 years (adults) (Table 5).

**Table 5:** Percentage improvement in FIM scores after cell transplantation based on different factors influencing the outcome of intervention

		No. of patients showing improved FIM	Percentage improvement
**Age at intervention**	Less than 18 years (*n* = 13)	10	76.92
	Above 18 years (*n* = 37)	20	54.05
**Multiple Doses**	Single dose (*n* = 35)	21	60
	Two doses (*n* = 15)	9	60
**Time since injury**	0–5 Years (*n* = 32)	21	65.62
	5 Years and above (*n* = 18)	9	50
**Severity based on GCS**	Mild (*n* = 27)	17	62.96
	Moderate (*n* = 16)	9	56.25
	Severe (*n* = 7)	4	57.14

#### Multiple doses

In our study, 15 patients underwent second dose of cell transplantation. On analysing the outcome on FIM, there was no difference observed in the group which underwent single dose as compared to the group of patients who underwent a second dose of transplantation (Table 5).

#### Time since injury

On analysing the effect of time since injury, it was found that patients who underwent cell transplantation within 5 years from their injury showed better outcome on FIM scale (Table 5).

#### Severity of injury based on GCS

On analysing the effect of severity of injury on the outcome of intervention, it was found that patients who were categorised as mild TBI based on CGS showed better outcome as compared to those who were moderate and severe TBI (Table 5).

### Adverse events

During the hospital stay, only 1 patient complained of headache and vomiting. There were no anesthesia related side effects witnessed in any of the patients. Within 3 months of the intervention, 2 patients had an episode of seizure as an adverse event, which was managed with medications. Both patients had abnormal EEG and 1 out of 2 had previous history of seizures and was on antiepileptics.

## Discussion

Chronic TBI is a complex and heterogeneous disease process ranging from the initial damage at the time of trauma to the consecutive destructive secondary damage (Hartings et al., 2009).Cell transplantation has shown potential to repair neuronal damage occurring in chronic TBI through neurorestorative and neuroprotective mechanisms. It utilizes the neurogenic capacity of stem cells to help in restoration of the damaged brain (Tajiri et al., 2014). The regenerative capacity of stem cells has been established in preclinical studies of TBI (Tajiri et al., 2014; Lee et al., 2010; Guo et al., 2017; Anbari et al., 2014; Acosta et al., 2015). Various studies involving animal models of TBI have shown improved motor function, cognitive function and behavioural outcome after cell transplantation using human fetal and embryonic-derived NSCs, blood, umbilical cord, placental, bone marrow progenitor, and mesenchymal stem cells. These improvements occur due to secretion of neurotrophic factors by these cells along with increased angiogenesis and reduced astrogliosis (Wennersten et al., 2004; Yan et al., 2013; Kim et al., 2010). Safety and feasibility of cell transplantation has also been established clinically. Zhang et al. studied the safety of bone marrow derived mesenchymal stem cells in 7 patients with TBI. They found that a combined procedure of intracranial and intravenous injection of autologous mesenchymal cells was safe and resulted in significantly improved neurological functions (Zhang et al., 2008). Similarly, Cox et al. and Liao et al. reported intravenous administration of autologous bone marrow mononuclear cells in children with severe traumatic brain injury to be safe with positive outcomes (Cox Jr et al., 2011; Liao et al., 2015). Our previously published pilot study also demonstrated safety and feasibility of autologous bone marrow mononuclear cell transplantation in 14 chronic TBI patients (Sharma et al., 2015). To further assess efficacy in larger population and for a long-term follow up, we conducted the presented study.

### Mechanism of action of bone marrow mononuclear cells in chronic TBI

Clinical application of bone marrow mononuclear cells (BMMNCs) is relatively safe and feasible (Zhang et al., 2013; Zhang et al., 2008; Savitz et al., 2011; Nguyen et al., 2017). They are a comprehensive cell population of various cell types like lymphocytes, monocytes of hematopoietic lineage, progenitor cells, mesenchymal cells, very small embryonic like (VSELs) stem cells and endothelial progenitor cells (EPCs) (Natividad Cuende et al., 2012). It has been observed that use of BMMNCs is more successful than the sub fractionated cell preparations (Lawall et al., 2010). The implanted BMMNCs follow the chemotactic pathway of SDF-1/CXCR4 and migrate to the site of injury and promote functional recovery (Li et al., 2015). BMMNCs multiply and differentiate into various cells such as neural cells and oligodendrocytes and promote neuroregeneration (Sanchez-Ramos et al., 2000). In TBI, myelin loss and axonal damage causes disruption of signal transduction. The oligodendrocytes help in remyelination and consequently repair the neural connections (Xu et al., 2015). BMMNCs protect the white matter degeneration and capillary breakdown secondary to acute injury (Park et al., 2014). They secrete various trophic growth factors such as nerve growth factor (NGF), glial derived neurotrophic factor (GDNF), brain derived neurotrophic factor (BDNF), vascular endothelial growth factor (VEGF), basic fibroblast growth factor (bFGF), which help in neuroprotection and inhibition of neuronal toxicity (Chen et al., 2002). These strategies limit secondary tissue loss and improve functional outcome in patients.

TBI also triggers a cascade of immunological and inflammatory tissue responses including depolarization of nerve terminal membrane and excessive release of excitatory neurotransmitters (Werner & Engelhard, 2007). The initial inflammatory response after TBI results in neuronal injury and activation of microglia resulting in the production of proinflammatory cytokines and neurotoxic chemicals (Hernandez-Ontiveros et al., 2013; Town et al., 2005). Cell transplantation simulates brain repair processes and has an anti-inflammatory and neuroprotective effect on the damaged cells (Shoichet et al., 2008). It has the potential to curtail the neuronal cell death and the prolonged secondary inflammatory response, which results in increased recovery in both cognitive and motor functions (Acosta et al., 2014; De la Pena et al., 2014). Bone marrow cells also secrete exosomes which are extracellular vesicles. Their therapeutic significance has been studied in animal models of TBI (Zhang et al., 2015). Exosomes may aid in functional recovery by inducing neurogenesis, angiogenesis and reducing inflammation (Feng et al., 2014). The consequent release of vasoconstrictors (prostaglandins and leukotrienes), microvascular dysfunction, the blood–brain barrier lesion, and the edema formation after TBI further reduces tissue perfusion and aggravates secondary brain damage (Prabhakar et al., 2014). The neuro-restorative effects exerted by the BMMNCs like angiogenesis, neovascularisation, production of growth factors, and paracrine effects enhances the tissue metabolism and thereby improves information processing in the damaged brain areas.

### Route of administration

The identification of the appropriate route of administration of cells is a crucial step, as the efficacy of intervention depends on the effective delivery of cells to the site of lesion. Direct intracerebral injection is an invasive procedure which may cause secondary damage (Abe et al., 2012). Intravenous transplantation is a minimally invasive procedure, however, the transplanted cells get trapped in the pulmonary passage and only few cells reach the target tissue (Fischer et al., 2009). Intrathecal route of administration provides the maximum homing capacity to the injected cells at the affected brain areas (Chen et al., 2015).

### Role of rehabilitation

As a part of the protocol, all the patients were given standard rehabilitation along with cell transplantation. It augments the therapeutic potential of the intervention. Enhanced proliferation of cells and increased mobilization of hematopoetic cells and erythropoetic cells in peripheral blood are some of the mechanisms triggered due to exercise (van Praag et al., 1999). Increase in angiogenesis and thus oxygen supply to the damaged areas of the brain is another aspect of exercise that improves the cognitive function. Regular exercise upregulates the chemokine guided SDF1/CXCR4+ pathway of homing and migration of stem cells and exerts an anti-inflammatory effect on the body (Bherer et al., 2013; Gomez-Pinilla & Hillman, 2013).

### Clinical outcome

Overall 92% patients showed improvements in various symptoms such as balance, voluntary control, memory, oromotor skills, lower limb activities, ambulation, trunk & upper limb activity, muscle tone, coordination, speech, posture, communication, psychological status, cognition, attention and concentration and ADLs. FIM was used to assess the functional status of the patients based on the level of assistance they required for their ADLs. Motor and cognitive impairment are the two most common outcomes of TBI (Godbolt et al., 2014; Breceda & Dromerick, 2013). FIM effectively evaluates patient’s functional abilities extensively covering cognitive and motor domains (Shukla et al., 2011). In this study, 60% patients showed improved FIM score suggesting improvement in ADLs such as bowel and bladder control, transfers, locomotion, communication, social cognition and self-care activities. Studies have shown that functional recovery after cell transplantation and neurorehabilitation could be attributed to endogenous plasticity, formation of new synapses and neural circuits which reorganize the damaged areas of the brain (Li & Chopp, 2009). New learning helps the brain establish new pathways and connections which enhances the information processing in these patients.

### Findings of PET CT scan brain

We used PET CT to monitor the outcome of cellular transplantation. Only 10 patients consented to perform a repeat PET CT for comparative analysis. [18F] Fludeoxyglucose (FDG PET) is a valuable tool in monitoring changes associated with early metabolic dysfunction and cognitive deficits in TBI. PET imaging provides exquisite sensitivity and information regarding changes in brain metabolism after TBI. Few studies have explored FDG-PET in TBI with varying degrees of sensitivity to detection at acute, subacute, and chronic phases of injury. These studies have shown reduced metabolism in the frontal, temporal, thalamic and cerebellar regions after the trauma (Granger et al., 1986; Giza & Hovda, 2014; Nakashima et al., 2007). These areas of the brain are directly responsible for behavioural and cognitive deficits in these patients. In this study, reduced metabolism was seen in frontal, temporal, parietal, mesial temporal, occipital, basal ganglia and cerebellar regions after the accident. These areas showed improved brain metabolism in the 10 patients who underwent PET CT scan brain 6 months after cell transplantation. These improvements correlated to the symptomatic changes observed in the patients at follow up. Similar results were published by Vaquero et al., wherein they demonstrated improved brain metabolism in the frontal, temporal, parietal, basal ganglia and cerebellar regions after intrathecal administration of autologous mesenchymal stromal cells which further resulted in clinical improvement in patients with diffuse axonal injury (Vaquero et al., 2017).

### Factors affecting the clinical outcomes

Various factors can affect the outcome of cell transplantation in chronic TBI. We analyzed the factors such as age, multiple doses of transplantation, time since injury and severity of injury, to study the effect of these on the efficacy and outcome of cellular transplantation.

#### Age at intervention

To study the effect of aging on the recovery of the chronic TBI patients after the cell transplantation, we compared the outcome on the objective scales in pediatric (< 18 years) and adult (≥ 18 years) patients.

It was observed that the pediatric patients demonstrated better improvements in the objective scale than the older patients (≥ 18 years). This outcome could be a result of the fact that brain plasticity is greater in the younger age and is malleable. The neurotransmitter interactions in neural circuit may also change as a function of age. For e.g. glutamate which has an important role in the maintenance of cellular function and apoptosis, reduces with age (Diaz-Arrastia et al., 2014). Intervention after injury at a younger age is likely to be more effective and can change the recovery trajectory in chronic TBI patients (Mahncke et al., 2006; Hebbeler et al., 2007).

#### Number of doses

To evaluate the efficiency of multiple transplantations, we performed a comparative analysis between patients who underwent single and two doses of BMMNC transplantation. In the present study, 60% of patients from both the groups showed improvement in FIM score. The number of doses did not affect the outcome of transplantation.

#### Time since injury

76.66% patients who underwent cell transplantation within 5 years of their injury showed improvement in their FIM score as compared to those who took intervention after 5 years of their injury. This could be because when the chronicity of the injury increases, it makes injury repair more difficult.

#### Severity based on GCS

Patients were categorised on severity based on the GCS. In this study, the outcome of intervention in patients with mild TBI was better as compared to moderate and severe TBI. 62.96% patients with mild TBI showed change in their FIM scores which indicate better functional recovery.

### Adverse events monitoring

In this study, all the patients were monitored for procedure related and cell transplantation related adverse events during their hospital stay as well as after discharge.

#### Procedure related adverse events

The adverse events associated with bone marrow aspiration and/or injection via lumbar puncture were categorized as procedure related adverse events. These included spinal headache, nausea, diarrhea, vomiting, pain or bleeding at the site of aspiration /injection, fever amongst others. Patients were also checked for anesthetic complications and allergic reactions. Only 1 patient complained of headache which was treated using medications.

#### Cell transplantation related adverse events

Two patients had an episode of seizures which was managed with medication. They both had an abnormal EEG before the intervention and 1 of them had previous history and was on antiepileptics. Occurrence of seizures did not affect the outcome of the intervention in these patients. The episode of seizures could be due to abnormal innervation in the neural circuits or activation of the epileptogenic foci during the process of neurogenesis (Banerjee et al., 2014). The longest follow up of this study population was 71 months and there were no cell related adverse events recorded indicating long-term safety.

### Limitations

One of the major limitations of the study was lack of control group to study the effect of cellular transplantation. However, all the chronic TBI patients were on rehabilitation before the intervention and their improvements were plateaued and did not show functional recovery. But after cellular transplantation along with rehabilitation, the patients showed significant improvement indicating that cell transplantation plays a vital role in the improvements. PET-CT scan was used as a monitoring tool in a small number of patients.

## Conclusion

This study demonstrates the safety and efficacy of cell transplantation in chronic TBI on long term follow up. Early intervention of cell transplantation at young age in patients with mild TBI showed the best outcome in this study. When combined with neurorehabilitation, cell transplantation may help to improve the quality of life of patients with chronic TBI by recovering their lost functions and making them independent in activities of daily living. The clinical improvements seen in the patients correlated with the metabolic improvements on PET CT scan post cell transplantation. Hence, PET CT scan may be used to monitor the changes in the brain functions after cell transplantation.

## Data Availability

Please contact author for data requests.

## References

[CR1] Abe K, Yamashita T, Takizawa S, Kuroda S, Kinouchi H, Kawahara N (2012). Stem cell therapy for cerebral ischemia: from basic science to clinical applications. J Cereb Blood Flow Metab.

[CR2] Acosta SA, Tajiri N, de la Pena I, Bastawrous M, Sanberg PR, Kaneko Y, Borlongan CV (2015). Alpha-synuclein as a pathological link between chronic traumatic brain injury and Parkinson's disease. J Cell Physiol.

[CR3] Acosta SA, Tajiri N, Hoover J, Kaneko Y, Borlongan CV (2015). Intravenous bone marrow stem cell grafts preferentially migrate to spleen and abrogate chronic inflammation in stroke. Stroke..

[CR4] Acosta SA, Tajiri N, Shinozuka K (2014). Combination therapy of human umbilical cord blood cells and granulocyte colony stimulating factor reduces histopathological and motor impairments in an experimental model of chronic traumatic brain injury. PLoS One.

[CR5] Anbari F, Khalili MA, Bahrami AR (2014). Intravenous transplantation of bone marrow mesenchymal stem cells promotes neural regeneration after traumatic brain injury. Neural Regen Res.

[CR6] Banerjee J, Chandra SP, Kurwale N, Tripathi M (2014). Epileptogenic networks and drug-resistant epilepsy: present and future perspectives of epilepsy research-utility for the epileptologist and the epilepsy surgeon. Ann Indian Acad Neurol.

[CR7] Bennett MH, Trytko B, Jonker B (2012). Hyperbaric oxygen therapy for the adjunctive treatment of traumatic brain injury. Cochrane Database Syst Rev.

[CR8] Bherer L, Erickson KI, Liu-Ambrose T (2013). A review of the effects of physical activity and exercise on cognitive and brain functions in older adults. J Aging Res.

[CR9] Breceda EY, Dromerick AW (2013). Motor rehabilitation in stroke and traumatic brain injury: stimulating and intense. Curr Opin Neurol.

[CR10] Chen BK, Knight AM, Nesbitt JJ, Butler GW, Padley DJ, Parisi JE, Dietz AB, Windebank AJ, Staff NP (2015). A safety study on intrathecal delivery of autologous mesenchymal stromal cells in rabbits directly supporting phase I human trials. Transfusion..

[CR11] Chen X, Katakowski M, Li Y, Lu D, Wang L, Zhang L, Chen J, Xu Y, Gautam S, Mahmood A, Chopp M (2002). Human bone marrow stromal cell cultures conditioned by traumatic brain tissue extracts: growth factor production. J Neurosci Res.

[CR12] Cox CS, Baumgartner JE, Harting MT, Worth LL, Walker PA, Shah SK, Ewing-Cobbs L, Hasan KM, Day MC, Lee D, Jimenez F, Gee A (2011). Autologous bone marrow mononuclear cell therapy for severe traumatic brain injury in children. Neurosurgery..

[CR13] De la Pena I, Sanberg PR, Acosta S, Lin SZ, Borlongan CV (2014). Umbilical cord blood cell and granulocyte-colony stimulating factor: combination therapy for traumatic brain injury. Regen Med.

[CR14] Diaz-Arrastia R, Kochanek PM, Bergold P, Kenney K, Marx C, Grimes J (2014). Pharmacotherapy of traumatic brain injury: state of the science and the road forward report of the department of defense neurotrauma pharmacology workgroup. J Neurotrauma.

[CR15] Feng Y, Lu SH, Wang X, Cui JJ, Li X, WJ DU, Wang Y, Li JJ, Song BQ, Chen F, Ma FX, Chi Y, Yang SG, Han ZC (2014). Biological characteristics of exosomes secreted by human bone marrow mesenchymal stem cells. Zhongguo Shi Yan Xue Ye Xue Za Zhi.

[CR16] Fischer UM, Harting MT, Jimenez F, Monzon-Posadas WO, Xue H, Savitz SI, Laine GA, Cox CS (2009). Pulmonary passage is a major obstacle for intravenous stem cell delivery: the pulmonary first-pass effect. Stem Cells Dev.

[CR17] Fleminger S, Ponsford J (2005). Long term outcome after traumatic brain injury: more attention needs to be paid to neuropsychiatric functioning. BMJ: Br Med J.

[CR18] Giza CC, Hovda DA (2014). The new neurometabolic cascade of concussion. Neurosurgery.

[CR19] Godbolt AK, Cancelliere C, Hincapie CA, Marras C, Boyle E, Kristman VL (2014). Systematic review of the risk of dementia and chronic cognitive impairment after mild traumatic brain injury: results of the international collaboration on mild traumatic brain injury prognosis. Arch Phys Med Rehabil.

[CR20] Gomez-Pinilla F, Hillman C (2013). The influence of exercise on cognitive abilities. Compr Physiol.

[CR21] Granger CV, Hamilton BB, Keith RA (1986). Advances in functional assessment for medical rehabilitation. Topics Geriatr Med.

[CR22] Guo S, Zhen Y, Wang A (2017). Transplantation of bone mesenchymal stem cells promotes angiogenesis and improves neurological function after traumatic brain injury in mouse. Neuropsychiatr Dis Treat.

[CR23] Hartings JA, Strong AJ, Fabricius M, Manning A (2009). Spreading depolarizations and late secondary insults after traumatic brain injury. J Neuro-Oncol.

[CR24] Hebbeler K, Spiker D, Bailey D, Scarborough A, Mallik S, Simeonsson R, Singer M (2007). Early intervention for infants & toddlers with disabilities and their families: participants, services, and outcomes.

[CR25] Hernandez-Ontiveros DG, Tajiri N, Acosta S, Giunta B, Tan J, Borlongan CV (2013). Microglia activation as a biomarker for traumatic brain injury. Front Neurol.

[CR26] Kim HJ, Lee JH, Kim SH (2010). Therapeutic effects of human mesenchymal stem cells on traumatic brain injury in rats: secretion of neurotrophic factors and inhibition of apoptosis. J Neurotrauma.

[CR27] Lawall H, Bramlage P, Amann B (2010). Stem cell and progenitor cell therapy in peripheral artery disease. A critical appraisal. Thromb Haemost.

[CR28] Lee JA, Kim BI, Jo CH, Choi CW, Kim EK, Kim HS, Yoon KS, Choi JH (2010). Mesenchymal stem-cell transplantation for hypoxic-ischemic brain injury in neonatal rat model. Pediatr Res.

[CR29] Li J, Guo W, Xiong M, Han H, Chen J, Mao D, Tang B, Yu H, Zeng Y (2015). Effect of SDF-1/CXCR4 axis on the migration of transplanted bone mesenchymal stem cells mobilized by erythropoietin toward lesion sites following spinal cord injury. Int J Mol Med.

[CR30] Li Y, Chopp M (2009). Marrow stromal cell transplantation in stroke and traumatic brain injury. Neurosci Lett.

[CR31] Liao GP, Harting MT, Hetz RA, Walker PA, Shah SK, Corkins CJ, Hughes TG, Jimenez F, Kosmach SC, Day MC, Tsao K, Lee DA, Worth LL, Baumgartner JE, Cox CS (2015). Autologous bone marrow mononuclear cells reduce therapeutic intensity for severe traumatic brain injury in children. Pediatr Crit Care Med.

[CR32] Liu S, Qu Y, Stewart TJ, Howard MJ, Chakrabortty S, Holekamp TF, McDonald JW (2000). Embryonic stem cells differentiate into oligodendrocytes and myelinate in culture and after spinal cord transplantation. Proc Natl Acad Sci.

[CR33] Mahncke HW, Bronstone A, Merzenich MM (2006). Brain plasticity and functional losses in the aged: scientific bases for a novel intervention. Prog Brain Res.

[CR34] McKee AC, Daneshvar DH (2015). The neuropathology of traumatic brain injury. Handb Clin Neurol.

[CR35] Muotri AR (2010). Pluripotent stem cells and neurological diseases. Estudos Avançados.

[CR36] Nakashima T, Nakayama N, Miwa K, Okumura A, Soeda A, Iwama T (2007). Focal brain glucose hypometabolism in patients with neuropsychologic deficits after diffuse axonal injury. Am J Neuroradiol.

[CR37] Natividad Cuende A, Laura Rico A, Concha Herrera B (2012). Concise Review: Bone Marrow Mononuclear Cells for the Treatment of Ischemic Syndromes: Medicinal Product or Cell Transplantation?. Stem Cells Transl Med.

[CR38] Nguyen LT, Nguyen AT, Vu CD, Ngo DV, Bui AV (2017). Outcomes of autologous bone marrow mononuclear cells for cerebral palsy: an open label uncontrolled clinical trial. BMC Pediatr.

[CR39] Park KJ, Park E, Liu E, Baker AJ (2014). Bone marrow-derived endothelial progenitor cells protect post ischemic axons after traumatic brain injury. J Cereb Blood Flow Metab.

[CR40] Prabhakar H, Sandhu K, Bhagat H, Durga P, Chawla R (2014). Current concepts of optimal cerebral perfusion pressure in traumatic brain injury. J Anaesthesiol Clin Pharmacol.

[CR41] Prins M, Greco T, Alexander D, Giza CC (2013). The pathophysiology of traumatic brain injury at a glance. Dis Model Mech.

[CR42] Roozenbeek B, Maas AI, Menon DK (2013). Changing patterns in the epidemiology of traumatic brain injury. Nat Rev Neurol.

[CR43] Sanchez-Ramos J, Song S, Cardozo-Pelaez F, Hazzi C, Stedeford T, Willing A, Freeman TB, Saporta S, Janssen W, Patel N, Cooper DR, Sanberg PR (2000). Adult bone marrow stromal cells differentiate into neural cells in vitro. Exp Neurol.

[CR44] Savitz SI, Misra V, Kasam M, Juneja H, Cox CS, Alderman S, Aisiku I, Kar S, Gee A, Grotta JC (2011). Intravenous autologous bone marrow mononuclear cells for ischemic stroke. Ann Neurol.

[CR45] Sharma A, Gokulchandran N, Sane H, Badhe P, Kulkarni P, Lohia M, Nagrajan A, Thomas N (2013). Detailed analysis of the clinical effects of cell therapy for thoracolumbar spinal cord injury: an original study. J Neurorestoratol.

[CR46] Sharma A, Gokulchandran N, Sane H, Nagrajan A, Paranjape A, Kulkarni P, Shetty A, Mishra P, Kali M, Biju H, Badhe P (2013). Autologous bone marrow mononuclear cell therapy for autism – an open label proof of concept study. Stem Cell Int.

[CR47] Sharma A, Sane H, Badhe P, Kulkarni P, Chopra G, Lohia M, Gokulchandran N (2012). Autologous bone marrow stem cell therapy shows functional improvement in hemorrhagic stroke- a case study. Indian J Clin Pract.

[CR48] Sharma A, Sane H, Kulkarni P, Yadav J, Gokulchandran N, Biju H, Badhe P (2015). Cell therapy attempted as a novel approach for chronic traumatic brain injury–a pilot study. SpringerPlus..

[CR49] Shoichet MS, Tate CC, Baumann MD, LaPlaca MC (2008). Strategies for regeneration and repair in the injured central nervous system in: Reichert WM, editor. Indwelling neural implants: strategies for contending with the in vivo environment.

[CR50] Shukla D, Devi BI, Agrawal A (2011). Outcome measures for traumatic brain injury. Clin Neurol Neurosurg.

[CR51] Tajiri N, Duncan K, Antoine A, Pabon M, Acosta SA, de la Pena I, Hernadez-Ontiveros DG, Shinozuka K, Ishikawa H, Kaneko Y, Yankee E, McGrogan M, Case C, Borlongan CV (2014). Stem cell-paved biobridge facilitates neural repair in traumatic brain injury. Front Syst Neurosci.

[CR52] Town T, Nikolic V, Tan J (2005). The microglial “activation” continuum: from innate to adaptive responses. J Neuroinflammation.

[CR53] Ul Hassan A, Hassan G, Rasool Z (2009). Role of stem cells in treatment of neurological disorder. Int J Health Sci (Qassim).

[CR54] van Praag H, Christie BR, Sejnowski TJ, Gage FH (1999). Running enhances neurogenesis, learning, and long-term potentiation in mice. Proc Natl Acad Sci U S A.

[CR55] Vaquero J, Zurita M, Bonilla C, Fernández C, Rubio JJ, Mucientes J, Rodriguez B, Blanco E, Donis L (2017). Progressive increase in brain glucose metabolism after intrathecal administration of autologous mesenchymal stromal cells in patients with diffuse axonal injury. Cytotherapy..

[CR56] Wennersten A, Meier X, Holmin S (2004). Proliferation, migration, and differentiation of human neural stem/progenitor cells after transplantation into a rat model of traumatic brain injury. J Neurosurg.

[CR57] Werner C, Engelhard K (2007). Pathophysiology of traumatic brain injury. Br J Anaesth.

[CR58] World Medical Association (2013). World medical association declaration of Helsinki: ethical principles for medical research involving human subjects. JAMA.

[CR59] Xiong Y, Mahmood A, Chopp M (2009). Emerging treatments for traumatic brain injury. Expert Opin Emerg Drugs.

[CR60] Xiong Y, Mahmood A, Chopp M (2010). Angiogenesis, neurogenesis and brain recovery of function following injury. Curr Opin Investig Drugs.

[CR61] Xu L, Ryu J, Hiel H, Menon A, Aggarwal A, Rha E, Mahairaki V, Cummings BJ, Koliatsos VE (2015). Transplantation of human oligodendrocyte progenitor cells in an animal model of diffuse traumatic axonal injury: survival and differentiation. Stem Cell Res Ther.

[CR62] Yan ZJ, Zhang P, Hu YQ (2013). Neural stem-like cells derived from human amnion tissue are effective in treating traumatic brain injury in rat. Neurochem Res.

[CR63] Zhang R, Liu Y, Yan K, Chen L, Chen XR, Li P, Chen FF, Jiang XD (2013). Anti-inflammatory and immunomodulatory mechanisms of mesenchymal stem cell transplantation in experimental traumatic brain injury. J Neuroinflammation.

[CR64] Zhang Y, Chopp M, Meng Y, Katakowski M, Xin H, Mahmood A, Xiong Y (2015). Effect of exosomes derived from multipluripotent mesenchymal stromal cells on functional recovery and neurovascular plasticity in rats after traumatic brain injury. J Neurosurg.

[CR65] Zhang ZX, Guan LX, Zhang K, Zhang Q, Dai LJ (2008). A combined procedure to deliver autologous mesenchymal stromal cells to patients with traumatic brain injury. Cytotherapy..

[CR66] Zhao LR, Duan WM, Reyes M, Keene CD, Verfaillie CM, Low WC (2002). Human bone marrow stem cells exhibit neural phenotypes and ameliorate neurological deficits after grafting into the ischemic brain of rats. Exp Neurol.

